# Phenotype-genotype correlations among carbapenem-resistant Enterobacterales recovered from four Egyptian hospitals with the report of SPM carbapenemase

**DOI:** 10.1186/s13756-022-01061-7

**Published:** 2022-01-21

**Authors:** Neveen A. Abdelaziz

**Affiliations:** Department of Microbiology and Immunology, Faculty of Pharmacy, Ahram Canadian University, POB: 12451, Sixth of October City, Giza Egypt

**Keywords:** Carbapenemases, CRE, Correlation, *Klebsiella pneumoniae*, Co-existence, *Bla*_NDM_, Surveillance

## Abstract

**Background:**

Carbapenem-resistant Enterobacterales (CRE), currently listed by the World Health Organization (WHO) as top priority critical pathogens, are a major global menace to human health. In low- and middle-income countries (LMICs) the threat is mounting fueled by selective pressures caused by antibiotic abuse and inadequate diagnostic resources.

**Methods:**

This study phenotypically and genotypically characterized carbapenem resistance among 115 Enterobacterales isolates including 76 *Klebsiella (K.) pneumoniae,* 19 *Escherichia (E.) coli,* 14 *Shigella (S.) sonnei*, 5 *Enterobacter (E.) cloacae*, and 1 *Proteus (P.) mirabilis*.

**Results:**

Ninety-three isolates (80.9%) were carbapenem-resistant with an alarming 57.5% carbapenem non-susceptibility in isolates collected from the outpatient department. Molecular characterization of the carbapenemases (CPases) encoding genes showed that *bla*_NDM_ (80.5%) was the most prevalent; it was detected in 62 isolates (54 *K. pneumoniae,* 6 *E. coli* and 2 *S. sonnei*), followed by *bla*_VIM_ (36.4%) which was observed in 28 isolates (24 *K. pneumoniae*, 3 *E. coli* and 1 *E. cloacae*). Other CPases included *bla*_KPC_ (28.6%; in 20 *K. pneumoniae*, 1 *E. coli* and 1 *S. sonnei*), *bla*_OXA-48_ (26%; in 17 *K. pneumoniae*, 1 *E. coli*,1 *E. cloacae* and 1 *P. mirabilis*), *bla*_IMP_ (6.5%; in 5 *K. pneumoniae*) and *bla*_SPM_ (1.3%; in *K. pneumoniae*). Notably more than half of the Enterobacterales isolates (54.5%) co-harboured more than one CPase-encoding gene. Co-existence of *bla*_NDM_ and *bla*_VIM_ genes was the most dominant (31.2%), followed by association of *bla*_NDM_ and *bla*_KPC_ (24.7%), then *bla*_VIM_ and *bla*_KPC_ (13%). Moreover, the effects of different genotypes on meropenem MIC values were assessed, and a statistically significant difference between the genotype (Ambler classes A and B) and the genotype (Ambler classes B and D) was recorded.

**Conclusion:**

The current findings may serve for a better understanding of the context of CRE in Egypt, associated drivers and CPases.

**Supplementary Information:**

The online version contains supplementary material available at 10.1186/s13756-022-01061-7.

## Background

Antimicrobial resistance (AMR) poses a serious global threat [1]. Over the past two decades the problem exacerbated due to the substantial increase in global antibiotic consumption [2]. Trends showed that low- and middle-income countries (LMICs), Egypt included, were the main consumers, with a surge in the use of “last-resort” antibiotics like carbapenems and polymyxins [3]. As a consequence, carbapenem-resistant Enterobacterales (CRE) endemic and epidemic emergence has been widely reported in hospitals [4, 5] and infrequently in community settings [6, 7]. Production of CPases comprises the main mechanism of carbapenem resistance in Enterobacterales. This further emphasizes the criticality of CRE, since CPases-encoding genes are typically found on mobile genetic elements (plasmids, transposons, etc.) which facilitates their dissemination [8]. Moreover, studies have shown the association of these genes with other AMR encoding genes such as extended-spectrum beta-lactamases (ESBLs), genes mediating resistance to aminoglycosides and fluoroquinolones which results in the upsurge of multidrug-resistant phenotypes [9]. Since CPases-encoding genes in Enterobacterales show wide diversity according to the geographic location, choosing the appropriate antibiotic is highly dependent upon rapid and accurate diagnostic tests. For instance, therapeutic decisions involving beta-lactamase inhibitors may fail against CPases, such as oxacillinases and metallo-beta-lactamases (MBLs), which necessitates identifying CPases on both the phenotypic and genomic levels [10]. Accordingly, LMICs encounter huge challenges due to scarce financial resources required for capacity building of efficient clinical laboratories to perform molecular-based tests. Relying solely on phenotypic methods to routinely report carbapenem resistance poses several challenges. First, the delay encountered to obtain the results of conventional culture-based approaches, which may force clinicians to rely on local hospital susceptibility data, as well as regional and national surveillance studies to guide their empirical treatment decisions. Besides, the obtained susceptibility patterns do not distinguish between carbapenem resistance due to carbapenemases production and that caused by other combined mechanisms such as extended spectrum beta-lactamases, AmpC and reduced permeability. As a result, carbapenems may be indicated for treatment even though a CPase is not present, hence unfavourable outcomes of potential antimicrobial resistance may arise. In Egypt, several studies were conducted to explore the prevalence of CRE strains among inpatients, nonetheless data concerning community acquired carbapenem resistance is lacking [11]. Considering all these challenges, we aimed to catch a glimpse of phenotype-genotype correlations among carbapenem-resistant Enterobacterales isolates from four Egyptian hospitals. We investigated the dissemination and the co-occurrence of CPases-encoding genes among the collected CRE isolates. Additionally, we tested the correlation between the identified genotypes and features such as the isolated organism, specimen source and department of isolation.

## Materials and methods

### Collection of clinical isolates

In this study, a total of 115 Enterobacterales isolates were obtained from the diagnostic microbiology laboratories of four Egyptian hospitals, namely Tanta University Hospital (TUH) (*n* = 47), National Cancer Institute (NCI) (*n* = 11), Memorial Souad Kafafi University Hospital (MSKUH) (*n* = 18), and El-Demerdash Hospital (DH) (*n* = 39) between March 2018 and September 2019. The isolates were recovered from clinical specimens, intended for routine microbiological diagnosis, collected from adult inpatients and outpatients. Only pus, blood, sputum, urine, and stool specimens were included. To avoid biased susceptibility results, we only included one Enterobacterales isolate per patient irrespective of the specimen type. Sputum and urine cultures were the major source of the recovered Enterobacterales isolates (47.8% and 43.5%, respectively), followed by pus (4.3%), stool (2.6%) and blood specimens (1.7%). Enterobacterales species were identified by traditional methods and confirmed using the MicroscanR WalkAway-96 Plus auto-identification system (Beckman Coulter, Miami, FL, USA) [12]. The collected 115 isolates included 76 (66.1%) *K. pneumoniae,* 19 (16.5%) *E. coli,* 14 (12.2%) *S. sonnei*, 5 (4.3%) *E. cloacae*, and 1 (0.9%) *P. mirabilis*. The isolates were mainly from inpatients 75 (65.2%) but also included outpatients 40 (34.8%). For everyday work, the isolates were stored at 4 °C in bacterial media, and for longer storage, they were stored in glycerol stock solutions at − 70 °C [13].

### Carbapenem susceptibility testing and MIC determination

The susceptibilities of the Enterobacterales isolates to imipenem (IPM) and meropenem (MER) were determined by the disc diffusion method according to the Clinical and Laboratory Standards Institute (CLSI) guidelines [14]. The susceptibility patterns of the isolates were interpreted according to the CLSI M07-A11 protocol [15]. Isolates were considered as CRE if they were found resistant or intermediate to one or both carbapenems (IPM and MER). Micro-broth dilution method was used to determine the minimum inhibitory concentration (MIC) of meropenem (AstraZeneca, UK) according to the CLSI guidelines [14]. Negative control wells contained only culture media to ensure sterility, while positive controls were inoculated with the organism, and *E. coli* ATCC 25922 was used as a reference strain. Following a 24 h incubation at 37 °C, the MIC readings were recorded and interpreted according to the CLSI M07-A11 protocol [15].

### PCR amplification of carbapenemases genes

Bacterial DNA was extracted using GeneJet™ DNA extraction kit (Thermo Fisher Scientific, Waltham, MA, USA) according to the manufacturer’s instructions and stored at − 20 °C for future use. PCR was performed using previously described primers (Thermo Fisher Scientific, Waltham, MA, USA) to amplify carbapenemases-encoding genes from Ambler class A: *bla*_KPC_ [16], class B metallo-beta-lactamases (MBLs): *bla*_VIM_, *bla*_IMP_, *bla*_SPM_ [17], *bla*_NDM_ [16], and class D oxacillinases: *bla*_OXA-48-like_ [16]. To easily interpret the expected bands and to avoid obtaining amplification products of the same size in each PCR tube, 2 multiplex reactions were performed for the detection of the 6 genes under investigation. The first multiplex reaction involved the detection of *bla*_KPC_, *bla*_SPM_ and *bla*_OXA-48-like_, while the second involved the detection of *bla*_VIM_, *bla*_IMP_ and *bla*_NDM_. Amplification conditions were initial denaturation at 94 °C for 5 min; 35 cycles of denaturation at 94 °C for 30 s, annealing at 58 °C-touch-down-52 °C for 30 s, and elongation at 72 °C for 30 s; then a final extension at 72 °C for 10 min in a TECHNE thermocycler (Bibby Scientific Ltd, Staffordshire, UK). Gel electrophoresis was carried out using the ADVANCE electrophoresis system (Mupid-exu, Tokyo, Japan) at 5 V/cm on 1.5% agarose gel, stained with 0.5 μg/ml ethidium bromide and visualized under ultraviolet light.

### Statistical analysis

All analyses were carried out using R statistical platform (https://www.r-project.org) in R-studio version 1.4.1106. Several R-packages were used in data analysis and visualization including *readxl, ggplot2, polycor* and *lares.* Fisher’s Exact (FE) test of independence was employed to analyze the associations between nominal variables. Moreover, the correlation between these variables was performed using the Spearman's rank correlation and strength of the association was expressed as Spearman's correlation coefficient (*r*_*s*_) between − 1 and + 1. Kruskal–Wallis (KW) test was used to compare the medians for non-normally distributed quantitative data and Mann–Whitney test was applied as a post-hoc test using Bonferroni correction method for multiple comparisons. For all statistical analyses, *p*-values ≤ 0.05 were considered statistically significant.

## Results

### CRE isolates

From a total of 115 Enterobacterales isolates, 80.9% (*n* = 93) were carbapenem-resistant and 19.1% (*n* = 22) were susceptible. As shown in Table 1, carbapenem resistance was highly enriched in *K. pneumoniae* (93.4%, *n* = 71) and *E. coli* (57.9%, *n* = 11) isolates (FE test *p* = 0.00001). As for the fourteen *S. sonnei* isolates, 6 (42.9%) were resistant to carbapenems with meropenem MICs ranging between 4 and 128 µg/ml. While 4 (80%) from five *E. cloacae* isolates and the *P. mirabilis* isolate were carbapenem-resistant with meropenem MICs ranging between 8 and 128 µg/ml (Additional file 1: Table 1S). Carbapenem-resistant isolates were obtained from the following specimens: sputum (57%, *n* = 53), urine (35.5%, *n* = 33), pus swab (5.4%, *n* = 5) and blood and stool (1.1%, *n* = 1, each). Isolates from the inpatient department displayed higher frequencies of carbapenem resistance (93.3%, *n* = 70/75) compared to those recovered from the outpatient department (57.5%, *n* = 23/40) (FE test *p* = 0.00001, Table 1).

**Table 1:** Association between carbapenem susceptibility and different features of 115 clinical Enterobacterales isolates

Features	No. of isolates (%)	Carbapenem susceptibility﻿	
		Resistant	Susceptible
**Organism**			
*Klebsiella pneumoniae*	76 (66.1%)	71	5
*Escherichia coli*	19 (16.5%)	11	8
*Shigella sonnei*	14 (12.2%)	6	8
*Enterobacter cloacae*	5 (4.3%)	4	1
*Proteus mirabilis*	1 (0.9%)	1	0
Fisher's Exact test *p*-value = 0.00001			
**Specimen**			
Sputum	55 (47.8%)	53	2
Urine	50 (43.5%)	33	17
Pus swab	5 (4.3%)	5	0
Stool	3 (2.6%)	1	2
Blood	2 (1.7%)	1	1
Fisher's Exact test *p*-value = 0.00003			
**Department of isolation**			
Inpatient	75 (65.2%)	70	5
Outpatient	40 (34.8%)	23	17
Fisher's Exact test *p*-value = 0.00001			

Organisms with < 30 isolates should be interpreted with caution, as small numbers may bias the group susceptibilities

### Molecular analysis of CPase genes

**Fig. 1:**
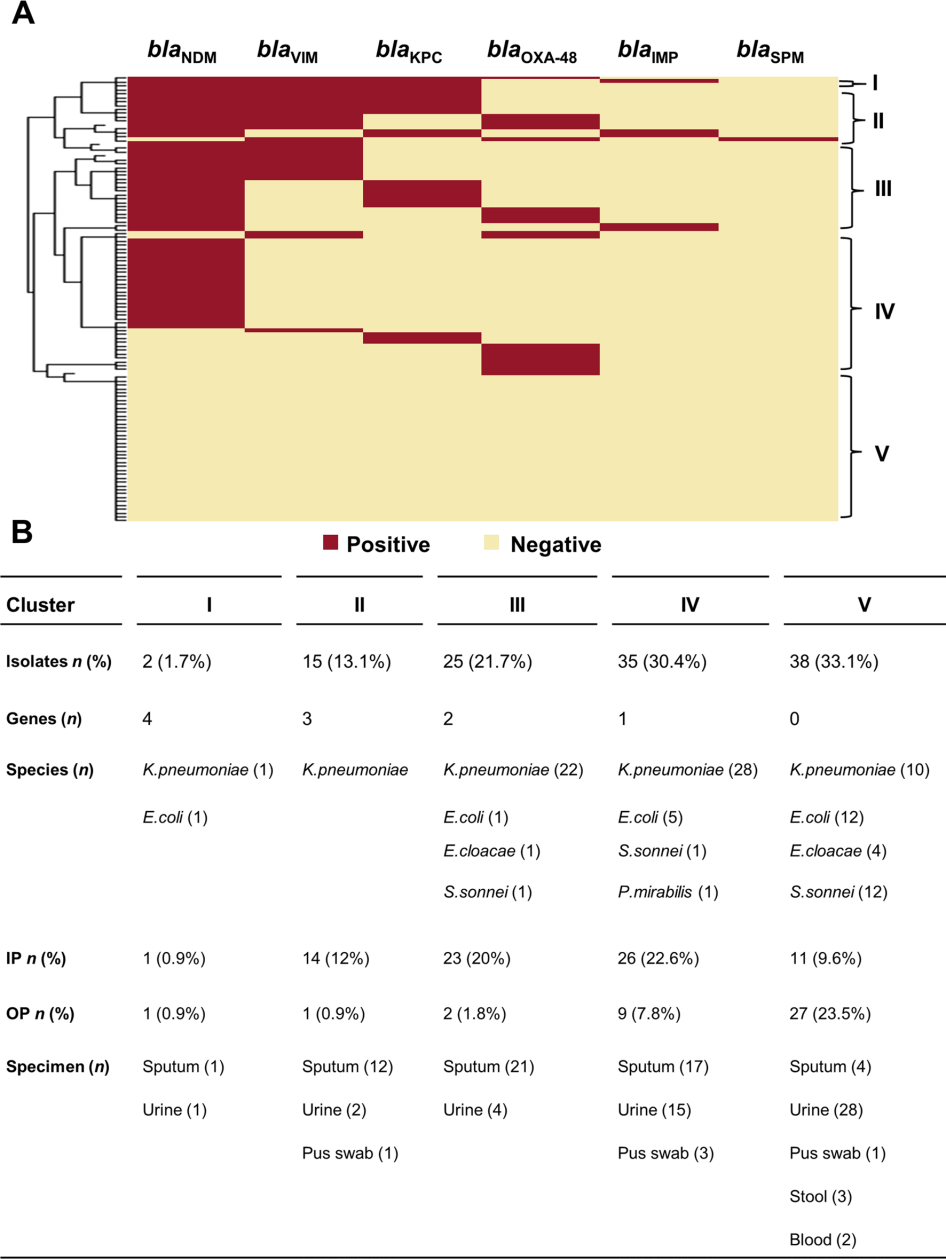
Distribution of screened CPases-encoding genes among the Enterobacterales isolates. **A** Differentiation of 115 Enterobacterales isolates into 5 clusters based on the observed number of CPases genes as per the colours in the figure key (PCR positive gene = red; PCR negative gene = yellow). **B** Tabular presentation of the characteristics of the clusters obtained in (**A**) showing detailed features of the identified genotypic patterns and the distribution among species, specimens, and department of isolation

The results of multiplex PCR assays are shown in Additional file 1: Table 1S. Among the 93 carbapenem-resistant Enterobacterales isolates, 82.8% (*n* = 77) harboured at least one or a combination of CPases-encoding genes, of these, 62 (80.5%) were *bla*_NDM_ positive (54 *K. pneumoniae,* 6 *E. coli* and 2 *S. sonnei*). Twenty-eight isolates (36.4%) expressed *bla*_VIM_ (24 *K. pneumoniae*, 3 *E. coli* and 1 *E. cloacae*), while 22 isolates (28.6%) expressed *bla*_KPC_ (20 *K. pneumoniae*, 1 *E. coli* and 1 *S. sonnei*). Twenty isolates (26%) exhibited *bla*_OXA-48_ (17 *K. pneumoniae*, 1 *E. coli*, 1 *E. cloacae* and 1 *P. mirabilis*). Finally, 5 isolates (6.5%), all *K. pneumoniae,* exhibited *bla*_IMP_ and only one exhibited *bla*_SPM_. For further evaluation of the identified genotypes, the isolates were clustered based on the observed number of CPases genes, and the resulting clusters were presented as a heat map (Fig. 1A). Clusters I, II, III and IV encompassed 77 isolates harbouring 4, 3,2 and 1 CPases genes, respectively. While cluster V included 38 isolates in which the tested genes were not detected. The details of the identified genotypes and the distribution among species, specimens, and department of isolation were presented in Fig. 1B. In *K. pneumoniae, bla*_NDM_ dominated (71.1%, *n* = 54), followed by *bla*_VIM_ (31.6%, *n* = 24), *bla*_KPC_ (26.3%, *n* = 20), *bla*_OXA-48_ (22.4%, *n* = 17), *bla*_IMP_ (6.6%, *n* = 5) and *bla*_SPM_ (1.3%, *n* = 1). A similar trend was observed in *E. coli* in which genes frequencies were *bla*_NDM_ (31.6%, *n* = 6), *bla*_VIM_ (15.8%, *n* = 3), *bla*_KPC_ and *bla*_OXA-48_ (5.3%, *n* = 1, each). Of the four carbapenem-resistant *E. cloacae* isolates included in this study, only one contained CPases genes (both *bla*_VIM_ and *bla*_OXA-48_). Likewise, only 2 out of the 6 carbapenem-resistant *S. sonnei* exhibited CPases genes, namely *bla*_NDM_ and *bla*_KPC_. Finally, the *P. mirabilis* isolate exhibited *bla*_OXA-48_ gene. Additionally, the effects of different genotypes on meropenem MICs in CPases positive isolates (*n* = 77) were assessed (Table 2). A Kruskal–Wallis test showed a statistically significant difference between the MICs against isolates with CPases-encoding genes belonging to different classes (*p-*value = 0.005328). Multiple comparisons using *post-hoc* Mann–Whitney test revealed a statistically significant difference between the genotype (Ambler classes A and B) and the genotype (Ambler classes B and D) (*p-*value = 0.000958). Notably, 16 isolates (5 *K. pneumoniae*, 4 *E. coli,* 4 *S. sonnei* and 3 *E. cloacae*) were phenotypically carbapenem-resistant but no CPases-encoding genes were detected.

**Table 2:** Distribution of Enterobacterales isolates (*n* = 77) according to minimum inhibitory concentration (MIC) of meropenem with the corresponding CPases-encoding genes

Ambler class CPases*	Meropenem MIC (µg/ml)								Median MIC (µg/ml)
	4	8	16	32	64	128	> 128	MIC_50_	
Class **A or B** carbapenemases	1	–	6	1	2	12	17	128	128
Classes **A and B** carbapenemases	1	–	–	1	–	3	13	> 128	256
Class **D** carbapenemases	–	–	1	2	1	2	2	64	96
Classes **B and D** carbapenemases	–	1	1	2	4	3	1	64	64

Kruskal–Wallis test H (3) = 12.7016*, p* = 0.005328

^*^Ambler class A: *bla*_KPC_, class B: *bla*_VIM_, *bla*_IMP_, *bla*_SPM_, *bla*_NDM_ and class D: *bla*_OXA-48-like_

### Co-existence of CPases-encoding genes

**Fig. 2:**
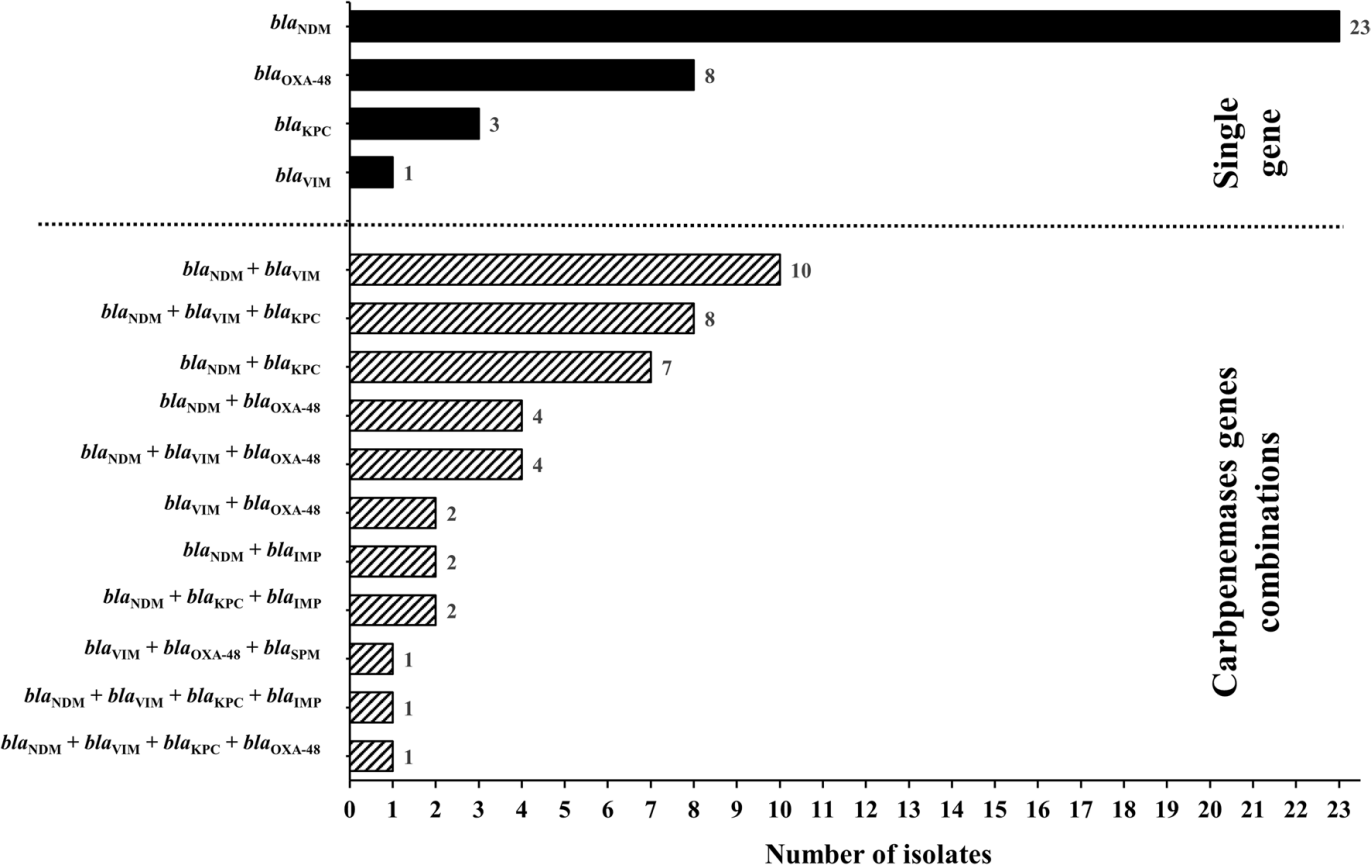
A bar graph summarizing the number of carbapenem-resistant Enterobacterales isolates (*n* = 77) harbouring CPases-encoding genes (*bla*_NDM_*, bla*_VIM_, *bla*_KPC_*, bla*_OXA-48_, *bla*_IMP_ and *bla*_SPM_) either in combination (stripped bars) or as single genes (black bars)

Remarkably, 42/77 (54.5%) Enterobacterales isolates co-harboured more than one CPase-encoding gene. In comparison to 35/77 (45.5%) ones expressing only a single gene (Fig. 2). A correlation matrix between the genes under investigation indicated that *bla*_NDM_ displayed the strongest correlations with *bla*_VIM_ and *bla*_KPC_ (*r*_*s*_ = 0.36 and 0.32, respectively) (Additional file 1: Fig. 1S). These results are in accordance with the recorded genotypic patterns (Fig. 2). Out of the 77 isolates harbouring CPases-encoding genes, the co-existence of *bla*_NDM_ and *bla*_VIM_ was detected in 24 isolates (31.2%) while expression of both *bla*_NDM_ and *bla*_KPC_ was recorded in 19 isolates (24.7%). Additional statistically significant correlations (*p-*value ≤ 0.05) were also reported between the following gene pairs; *bla*_VIM_ and *bla*_KPC_ (*r*_*s*_ = 0.24), *bla*_IMP_ and *bla*_KPC_ (*r*_*s*_ = 0.22), *bla*_OXA-48_ and *bla*_SPM_ (*r*_*s*_ = 0.2), *bla*_NDM_ and *bla*_IMP_ (*r*_*s*_ = 0.2) (Additional file 1: Fig. 1S).

### Features associated with the carriage of CPases-encoding genes

**Fig. 3:**
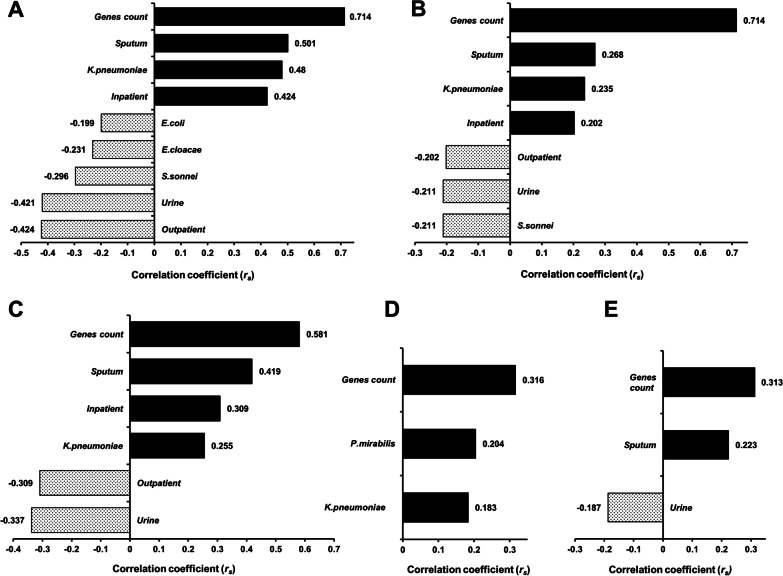
Features associated with the carriage of CPases-encoding genes, namely: **A** *bla*_NDM_; **B** *bla*_VIM_; **C** *bla*_KPC_; **D** *bla*_OXA-48_; **E** *bla*_IMP_. Only statistically significant (*p-*value ≤ 0.05) correlations were plotted. Positive correlations are designated a black colour and negative ones are dotted, and all are labelled with the corresponding Spearman’s rank correlation coefficient (*rs*) value. The figure was generated using “corr_var” function, “lares” package (version 4.7)

We explored the correlation between the detected *bla*_NDM_, *bla*_VIM_, *bla*_KPC_, *bla*_OXA-48_, *and bla*_IMP_ genes and the documented features of the isolates (Fig. 3). Interestingly, *bla*_NDM_, *bla*_VIM_, and *bla*_KPC_ (Fig. 3A–C, respectively) demonstrated statistically significant (*p-*value ≤ 0.05) positive correlations with the same features, namely, the count of genes detected in combination, *K. pneumoniae* isolates from sputum cultures, and recovery from inpatients. Likewise, the count of genes detected in combination was also positively correlated with both *bla*_OXA-48_ and *bla*_IMP_ (*r*_*s*_ = 0.316 and 0.313, respectively) (Fig. 3D, E, respectively). On the other hand, statistically significant negative correlations were obtained between certain features and the genes *bla*_NDM_, *bla*_VIM_, and *bla*_KPC_. For instance, isolates from urine samples obtained from outpatients were less likely to harbour *bla*_NDM_, *bla*_VIM_, and *bla*_KPC_ genes.

## Discussion

To tackle the AMR crisis, the WHO assembly adopted a global action plan with five main objectives [18]. One goal is to strengthen the knowledge and evidence base through surveillance and research. Surveillance data can monitor the drivers of AMR and track trends across multi-sectors [19, 20]. Consequently, interpretation of such data endorses the implementation of infection control programs [21, 22], besides guiding the choice of resistance testing methods and treatment decisions [10]. Egypt’s healthcare-associated infections (HAI) surveillance system, established in 2011, is a leading prospective, standardized system in WHO’s Eastern Mediterranean Region (EMR) [23, 24]. However, limited studies have reported the increasing burden of CRE in Egypt using the system’s surveillance data [5]. On the other hand, the academic network strived to provide research data to fill the knowledge gap regarding the severity of CRE problem in Egypt [11]. Routine testing for CRE resistance mechanisms is still not mandated in many clinical laboratories [25]. Consequently, they are unable to differentiate carbapenem non-susceptibility caused by carbapenemases production from resistance because of other mechanisms such as overexpression of AmpC enzymes and membrane impermeability. The present study aimed to detect CPases, the most important mechanism of carbapenem resistance, among 115 Enterobacterales isolates recovered from inpatients and outpatients in 4 major Egyptian hospitals. *K. pneumoniae* was the most frequently recovered isolate (66.1%), followed by *E. coli* (16.5%), *S. sonnei* (12.2%), *E. cloacae* (4.3%) and *P. mirabilis* (0.9%). In outpatients, *E. coli* and *S. sonnei* isolates dominated (43% and 30%, respectively). Strikingly, carbapenem resistance was detected in 80.9% of the Enterobacterales isolates. This level of resistance is higher than recent studies in Egypt describing the isolation of CRE with the percentages 46% [26], 59% [27], 68.8% [28] from cancer patients and 48% from intensive care units [5]. In the study performed by Hassuna et al. [29], very high frequency of carbapenem resistance (95%) was detected among the *K. pneumoniae* isolates. Comparable rates were observed in this study, in which *K. pneumoniae* cultured from sputum and pus specimens obtained from inpatients were highly enriched with carbapenem resistance 96–100%. In agreement with previous studies [30] we found significant differences in carbapenem resistance between Enterobacterales isolated from inpatients compared to outpatients, which may be due to the selective pressure of excessive consumption of antibiotics in hospitals. It has been estimated that 75% of patients treated mostly for respiratory infections receive empirical carbapenem therapy [31]. Furthermore, a study involving 97,910 strains of *K. pneumoniae* found a correlation factor of 0.427 (*p* < 0.01) between carbapenem consumption intensity and rate of carbapenem resistance [32]. In agreement with these observations, the high carbapenem resistance rates in this study underline a pressing need for a national antimicrobial stewardship plan in Egypt to optimize the utilization of carbapenems as treatment options, especially in respiratory tract infections. The WHO provides a practical implementation guidance for the recommendations targeting health care facilities regarding the prevention and control of CRE, comprising how to utilize surveillance results, and put in place antibiotic stewardship programmes [33].

Next, we molecularly characterized the CPases-encoding genes in our isolates. Amid the positive genotypes, *bla*_NDM_ (80.5%) was predominant, followed by *bla*_VIM_ (36.4%), *bla*_KPC_ (28.6%), *bla*_OXA-48_ (26%) and *bla*_IMP_ (6.5%). Similar percentages were recently reported in Egypt [29, 34, 35]. Even though, the *bla*_SPM_ was formerly detected in *Pseudomonas aeruginosa* isolates [36–38], here we report the first-time detection of the rare *bla*_SPM_ in combination with both *bla*_VIM_ and *bla*_OXA-48_ from a *K. pneumoniae* isolate in Egypt. Our results uncovered substantial co-existence of CPases-encoding genes (54.5%), this may be attributed to the fact that these genes are carried on mobile genetic elements and thus are easily transferred within different hospital settings [39]. Twenty-four isolates (31.2%) co-harboured *bla*_NDM_ and *bla*_VIM_ genes. This percentage is higher than in previous studies from Egypt by Kamel et al. [26] and Khalil et al. [40] in which such association was detected in only 1 (3%) and 4 (8.7%) isolates, respectively. Whereas more current investigations reported higher rates ranging between 69% [41] and 100% [29]. On the other hand, association of *bla*_NDM_ and *bla*_KPC_ was detected in 19 (24.7%) isolates which is comparable to results by Ramadan et al., (33.3%) [35], but higher than Ragheb et al., (5%) [41], Khalil et al., (8.7%) [40] and El-Kholy et al., (10%) [38]. Additionally, *bla*_VIM_ and *bla*_KPC_ were simultaneously present in 10 (13%) isolates, in agreement with previous reports with roughly the same incidences [40, 41]. Researchers have formerly assessed the effects of different genotypes on carbapenem MIC values [42]. Similarly, our results record a statistically significant difference between the genotype (Ambler classes A and B) and the genotype (Ambler classes B and D). This finding may be due to the low level hydrolytic activity of *bla*_OXA-48_ [43]. It is noteworthy that 16 (13.9%) isolates with phenotypic carbapenem-resistant profiles were negative for the tested CPases genes. In accordance with other studies [7] this may be attributed to types of carbapenemases different than those tested in this study. Besides, the presence of other combined mechanisms such as extended spectrum beta-lactamases, AmpC and reduced permeability to carbapenems caused by porin mutations [44, 45].

What is more alarming, is the excessive rates (57.5%) of carbapenem resistance in isolates collected from the outpatient department. Even though, 10 out of the 23 non-susceptible isolates were negative for the genes used in this study, yet the resistance level conferred by the positive CPases genes (32.5%) is worrisome. Regrettably, CRE strains could circulate in community settings and the resulting infections may easily propagate especially with the lack of proper hygiene measures [7]. Additionally, in many LMICs, including Egypt, patients can purchase antibiotics without a physician’s prescription [46]. In a recent study conducted among 563 Egyptian medical students, about 77.7% of the participants were self-medicated [47].

A major goal of this study was to detect features associated with carriage of CPases-encoding genes. Successful strategies for preventing the transmission of these problematic strains require thorough and meaningful characterization of these infections in respect to causative organism, department of isolation and specimen source. Such approach may prompt the improvement of the applied infection control measures, provide guidelines for effective empiric therapy, and regulate the use of novel combinations involving beta-lactamase inhibitors. In agreement with previous studies [10], *bla*_NDM_ and *bla*_KPC_ were strongly correlated to *K. pneumoniae* isolated from sputum cultures recovered from inpatients. Correspondingly, improved infection control regimens are needed, primarily with patients suffering from respiratory tract infections caused by *K. pneumoniae*. A recent study highlighted the association between contamination of hospital’s environment and transmission of carbapenem-resistant *K. pneumoniae* in ICUs [48]. Furthermore, long term carriage of *bla*_KPC_, for up to 3 years, was defined in patients discharged from a hospital with an outbreak of KPC producing *K. pneumoniae* [49]. Of note, all the investigated CPases-encoding genes, except *bla*_SPM_, were significantly associated with the overall count of genes detected in combination. This implies the high likelihood of the co-existence of the investigated CPases-encoding genes which raises deep concerns about the simultaneous transmission of such genes.

Even though sample-based surveillance enabled us to report the most frequent types of ﻿ infections caused by CRE, however this study had some limitations. First, the number of clinical isolates was small. Hence, it was insufficient to draw firm conclusions about the true resistance rates except for *K. pneumoniae* [50]. Second, isolates were obtained anonymously from the microbiology laboratories, so no data were available on the patient’s microbiological history and prior antibiotic use. Finally, with respect to outpatients, a common practice in Egypt is to seek antimicrobials without being tested for susceptibility, thus the examined isolates are more likely to be from patients who have failed first line and even second line treatments, which might have caused an overestimation of the resistance rates in this study.

## Conclusion

This study underlines the escalating dilemma of CRE prevalence in Egyptian hospitals and sheds the light on the rising incidences in community settings. Here we elucidated the prevalent CPases-encoding genes present either in combination or as single genes. To address this challenge, ongoing identification, and risk-based surveillance of CRE are indispensable. Equally crucial is the urgent implementation of a national antimicrobial stewardship plan, to diminish the current burden of CRE in Egyptian hospitals and circumvent its emergence in community settings.

## Supplementary Information

**Additional file 1.** Results of carbapenem susceptibility, multiplex PCR and correlations between CPases.

## Data Availability

All data generated or analysed during this study are included in this published article and supplementary file.

## Abbreviations

LMICs: Low- and middle-income countries
CRE: Carbapenem-resistant Enterobacterales
CPase: Carbapenemase
AMR: Antimicrobial resistance
ESBLs: Extended-spectrum beta-lactamases
MBLs: Metallo- beta-lactamases
IPM: Imipenem
MER: Meropenem
FE test: Fisher’s Exact test
KW test: Kruskal–Wallis test
IP: Inpatient
OP: Outpatient
WHO: World Health Organization
HAI: Healthcare-associated infection
EMR: Eastern Mediterranean Region
TUH: Tanta University Hospital
NCI: National Cancer Institute
MSKUH: Memorial Souad Kafafi University Hospital
DH: El-Demerdash Hospital
CLSI: Clinical Laboratory Standard Institute
