# Supplementary material for: Phenotype-genotype correlations among carbapenem-resistant Enterobacterales recovered from four Egyptian hospitals with the report of SPM carbapenemase
Source: Antimicrob Resist Infect Control. 2022 Jan 21;11:13. doi: 10.1186/s13756-022-01061-7 (PMC8783469; doi:10.1186/s13756-022-01061-7)
Supplement: Supplementary file 1 — Additional file 1. Results of carbapenem susceptibility, multiplex PCR and correlations between CPases. [file 13756_2022_1061_MOESM1_ESM.docx]

**Phenotype-genotype correlations among carbapenem-resistant Enterobacterales recovered from four Egyptian hospitals with the report of SPM carbapenemase**

**Neveen A. Abdelaziz**

Department of Microbiology and Immunology, Faculty of Pharmacy, Ahram Canadian University, Sixth of October City, Giza, Egypt.

**Correspondence:**Address: Department of Microbiology and Immunology, Faculty of Pharmacy, Ahram Canadian University, POB: 12451, Sixth of October City, Giza, Egypt.

E-mail: [neveen.abdelaziz@acu.edu.eg](mailto:neveen.abdelaziz@acu.edu.eg)

**Supplementary Table:**

**Table 1S:** Carbapenem susceptibility**,** Minimum inhibitory concentration (MIC), carbapenemases-encoding genes, and features of 115 Enterobacterales isolates

| **Isolate No.** | **Organism** | **Carbapenem susceptibility** | **MER MIC (μg/ml)** | **IP/OP** | **Specimen** | **Class A CPases** | **Class B CPases** | | | | **Class D CPases** |
| --- | --- | --- | --- | --- | --- | --- | --- | --- | --- | --- | --- |
|  |  |  |  |  |  | ***bla*_KPC_** | ***bla*_NDM_** | ***bla*_VIM_** | ***bla*_IMP_** | ***bla*_SPM_** | ***bla*_OXA-48_** |
| 60 | *K. pneumoniae* | R | 64 | IP | Pus swab | - | + | + | - | - | + |
| 62 | *K. pneumoniae* | R | 32 | IP | Sputum | - | + | + | - | - | + |
| 63 | *K. pneumoniae* | R | 128 | OP | Urine | - | + | + | - | - | + |
| 64 | *K. pneumoniae* | R | 128 | IP | Sputum | - | + | + | - | - | + |
| 86 | *K. pneumoniae* | R | 32 | IP | Sputum | + | + | + | + | - | - |
| 100 | *K. pneumoniae* | R | 128 | IP | Sputum | + | + | + | - | - | - |
| 102 | *K. pneumoniae* | R | >128 | IP | Sputum | + | + | + | - | - | - |
| 104 | *K. pneumoniae* | R | >128 | IP | Sputum | + | + | + | - | - | - |
| 105 | *K. pneumoniae* | R | >128 | IP | Sputum | + | + | + | - | - | - |
| 106 | *K. pneumoniae* | R | >128 | IP | Sputum | + | + | + | - | - | - |
| 109 | *K. pneumoniae* | R | 128 | IP | Sputum | + | + | + | - | - | - |
| 117 | *K. pneumoniae* | R | >128 | IP | Sputum | + | + | + | - | - | - |
| 118 | *K. pneumoniae* | R | >128 | IP | Sputum | + | + | + | - | - | - |
| 58 | *K. pneumoniae* | R | 16 | OP | Urine | - | + | + | - | - | - |
| 61 | *K. pneumoniae* | R | 128 | IP | Sputum | - | + | + | - | - | - |
| 103 | *K. pneumoniae* | R | >128 | IP | Sputum | - | + | + | - | - | - |
| 108 | *K. pneumoniae* | R | 128 | IP | Sputum | - | + | + | - | - | - |
| 112 | *K. pneumoniae* | R | >128 | IP | Sputum | - | + | + | - | - | - |
| 113 | *K. pneumoniae* | R | >128 | IP | Sputum | - | + | + | - | - | - |
| 114 | *K. pneumoniae* | R | >128 | IP | Sputum | - | + | + | - | - | - |
| 115 | *K. pneumoniae* | R | >128 | IP | Sputum | - | + | + | - | - | - |
| 116 | *K. pneumoniae* | R | >128 | IP | Sputum | - | + | + | - | - | - |
| 76 | *K. pneumoniae* | R | >128 | IP | Sputum | - | + | - | - | - | + |
| 77 | *K. pneumoniae* | R | 8 | IP | Sputum | - | + | - | - | - | + |
| 78 | *K. pneumoniae* | R | 32 | IP | Sputum | - | + | - | - | - | + |
| 79 | *K. pneumoniae* | R | 64 | IP | Sputum | - | + | - | - | - | + |
| 96 | *K. pneumoniae* | R | >128 | IP | Sputum | + | + | - | + | - | - |
| 97 | *K. pneumoniae* | R | >128 | IP | Sputum | + | + | - | + | - | - |
| 87 | *K. pneumoniae* | R | >128 | IP | Sputum | + | + | - | - | - | - |
| 89 | *K. pneumoniae* | R | >128 | IP | Sputum | + | + | - | - | - | - |
| 90 | *K. pneumoniae* | R | >128 | IP | Sputum | + | + | - | - | - | - |
| 91 | *K. pneumoniae* | R | 128 | IP | Sputum | + | + | - | - | - | - |
| 94 | *K. pneumoniae* | R | >128 | IP | Sputum | + | + | - | - | - | - |
| 99 | *K. pneumoniae* | R | >128 | IP | Sputum | + | + | - | - | - | - |
| 93 | *K. pneumoniae* | R | >128 | IP | Sputum | - | + | - | + | - | - |
| 98 | *K. pneumoniae* | R | >128 | IP | Sputum | - | + | - | + | - | - |
| 8 | *K. pneumoniae* | R | 16 | IP | Urine | - | + | - | - | - | - |
| 19 | *K. pneumoniae* | R | 64 | IP | Urine | - | + | - | - | - | - |
| 47 | *K. pneumoniae* | R | >128 | OP | Urine | - | + | - | - | - | - |
| 48 | *K. pneumoniae* | R | >128 | OP | Urine | - | + | - | - | - | - |
| 49 | *K. pneumoniae* | R | >128 | IP | Pus swab | - | + | - | - | - | - |
| 50 | *K. pneumoniae* | R | 128 | IP | Pus swab | - | + | - | - | - | - |
| 51 | *K. pneumoniae* | R | 128 | IP | Sputum | - | + | - | - | - | - |
| 52 | *K. pneumoniae* | R | 4 | OP | Urine | - | + | - | - | - | - |
| 80 | *K. pneumoniae* | R | 64 | IP | Sputum | - | + | - | - | - | - |
| 81 | *K. pneumoniae* | R | >128 | IP | Sputum | - | + | - | - | - | - |
| 82 | *K. pneumoniae* | R | 16 | IP | Sputum | - | + | - | - | - | - |
| 83 | *K. pneumoniae* | R | >128 | IP | Sputum | - | + | - | - | - | - |
| 84 | *K. pneumoniae* | R | >128 | IP | Sputum | - | + | - | - | - | - |
| 88 | *K. pneumoniae* | R | >128 | IP | Sputum | - | + | - | - | - | - |
| 92 | *K. pneumoniae* | R | 128 | IP | Sputum | - | + | - | - | - | - |
| 95 | *K. pneumoniae* | R | >128 | IP | Sputum | - | + | - | - | - | - |
| 110 | *K. pneumoniae* | R | 128 | IP | Sputum | - | + | - | - | - | - |
| 111 | *K. pneumoniae* | R | 128 | IP | Sputum | - | + | - | - | - | - |
| 23 | *K. pneumoniae* | R | 128 | IP | Urine | - | - | + | - | + | + |
| 12 | *K. pneumoniae* | R | 16 | IP | Urine | - | - | + | - | - | + |
| 13 | *K. pneumoniae* | R | 32 | IP | Urine | - | - | - | - | - | + |
| 17 | *K. pneumoniae* | R | 16 | IP | Urine | - | - | - | - | - | + |
| 45 | *K. pneumoniae* | R | >128 | IP | Sputum | - | - | - | - | - | + |
| 46 | *K. pneumoniae* | R | 64 | OP | Urine | - | - | - | - | - | + |
| 53 | *K. pneumoniae* | R | 128 | IP | Pus swab | - | - | - | - | - | + |
| 54 | *K. pneumoniae* | R | 32 | OP | Urine | - | - | - | - | - | + |
| 57 | *K. pneumoniae* | R | >128 | IP | Sputum | - | - | - | - | - | + |
| 85 | *K. pneumoniae* | R | 32 | IP | Sputum | + | - | - | - | - | - |
| 101 | *K. pneumoniae* | R | 128 | IP | Sputum | + | - | - | - | - | - |
| 107 | *K. pneumoniae* | R | >128 | IP | Sputum | + | - | - | - | - | - |
| 18 | *K. pneumoniae* | R | 128 | IP | Urine | - | - | - | - | - | - |
| 20 | *K. pneumoniae* | R | 64 | IP | Urine | - | - | - | - | - | - |
| 55 | *K. pneumoniae* | R | 64 | OP | Urine | - | - | - | - | - | - |
| 56 | *K. pneumoniae* | R | 32 | IP | Sputum | - | - | - | - | - | - |
| 119 | *K. pneumoniae* | R | 4 | OP | Stool | - | - | - | - | - | - |
| 22 | *K. pneumoniae* | S | 0.5 | OP | Stool | - | - | - | - | - | - |
| 26 | *K. pneumoniae* | S | 1 | IP | Urine | - | - | - | - | - | - |
| 59 | *K. pneumoniae* | S | 2 | OP | Urine | - | - | - | - | - | - |
| 74 | *K. pneumoniae* | S | 1 | IP | Sputum | - | - | - | - | - | - |
| 75 | *K. pneumoniae* | S | 0.5 | IP | Sputum | - | - | - | - | - | - |
| 72 | *E. coli* | R | 64 | OP | Urine | + | + | + | - | - | + |
| 66 | *E. coli* | R | 16 | OP | Urine | - | + | + | - | - | - |
| 2 | *E. coli* | R | 16 | IP | Urine | - | + | - | - | - | - |
| 4 | *E. coli* | R | 128 | IP | Urine | - | + | - | - | - | - |
| 65 | *E. coli* | R | 128 | OP | Urine | - | + | - | - | - | - |
| 70 | *E. coli* | R | 128 | OP | Urine | - | + | - | - | - | - |
| 31 | *E. coli* | R | 16 | OP | Urine | - | - | + | - | - | - |
| 34 | *E. coli* | R | 4 | OP | Urine | - | - | - | - | - | - |
| 67 | *E. coli* | R | 128 | OP | Urine | - | - | - | - | - | - |
| 68 | *E. coli* | R | 8 | OP | Urine | - | - | - | - | - | - |
| 73 | *E. coli* | R | 128 | OP | Urine | - | - | - | - | - | - |
| 27 | *E. coli* | S | 1 | OP | Urine | - | - | - | - | - | - |
| 28 | *E. coli* | S | 1 | OP | Urine | - | - | - | - | - | - |
| 29 | *E. coli* | S | 0.5 | OP | Urine | - | - | - | - | - | - |
| 33 | *E. coli* | S | 0.5 | OP | Urine | - | - | - | - | - | - |
| 42 | *E. coli* | S | 0.5 | OP | Urine | - | - | - | - | - | - |
| 69 | *E. coli* | S | 1 | OP | Urine | - | - | - | - | - | - |
| 71 | *E. coli* | S | 1 | OP | Urine | - | - | - | - | - | - |
| 121 | *E. coli* | S | 1 | OP | Urine | - | - | - | - | - | - |
| 7 | *S. sonnei* | R | 8 | IP | Urine | + | + | - | - | - | - |
| 32 | *S. sonnei* | R | 128 | OP | Urine | - | + | - | - | - | - |
| 30 | *S. sonnei* | R | 8 | OP | Urine | - | - | - | - | - | - |
| 37 | *S. sonnei* | R | 8 | OP | Urine | - | - | - | - | - | - |
| 39 | *S. sonnei* | R | 8 | OP | Urine | - | - | - | - | - | - |
| 40 | *S. sonnei* | R | 4 | OP | Urine | - | - | - | - | - | - |
| 3 | *S. sonnei* | S | 0.25 | IP | Blood | - | - | - | - | - | - |
| 14 | *S. sonnei* | S | 0.25 | OP | Stool | - | - | - | - | - | - |
| 35 | *S. sonnei* | S | 0.25 | OP | Urine | - | - | - | - | - | - |
| 36 | *S. sonnei* | S | 0.25 | OP | Urine | - | - | - | - | - | - |
| 38 | *S. sonnei* | S | 1 | OP | Urine | - | - | - | - | - | - |
| 41 | *S. sonnei* | S | 1 | OP | Urine | - | - | - | - | - | - |
| 43 | *S. sonnei* | S | 1 | OP | Urine | - | - | - | - | - | - |
| 44 | *S. sonnei* | S | 2 | OP | Urine | - | - | - | - | - | - |
| 21 | *E. cloacae* | R | 64 | IP | Sputum | - | - | + | - | - | + |
| 15 | *E. cloacae* | R | 128 | IP | Blood | - | - | - | - | - | - |
| 24 | *E. cloacae* | R | 64 | IP | Pus swab | - | - | - | - | - | - |
| 120 | *E. cloacae* | R | 8 | IP | Sputum | - | - | - | - | - | - |
| 10 | *E. cloacae* | S | 0.5 | IP | Urine | - | - | - | - | - | - |
| 16 | *P. mirabilis* | R | 128 | IP | Sputum | - | - | - | - | - | + |

**Supplementary Figure:**


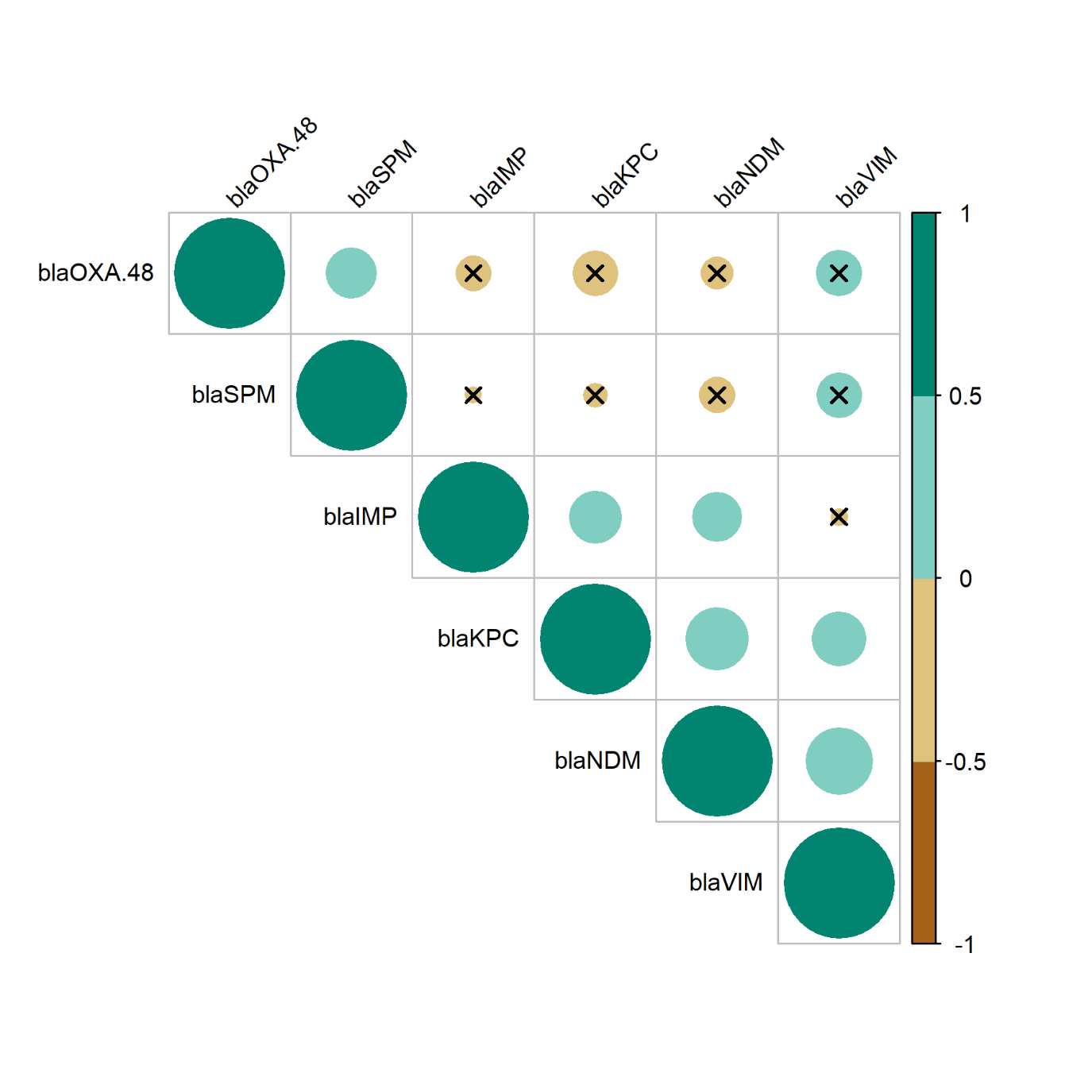
 **Figure 1S.** A Correlogram representing correlation coefficients between each pair of the investigated carbapenemases-encoding genes employing the multiplex PCR results of the 115 isolates. Non-statistically significant correlations are crossed out and only statistically significant ones (*p-*value ≤ 0.05) were considered. The colour intensity represents Spearman’s rank correlation coefficient (*rs*) value (Green tones are positive correlations and brown tones are negative ones).
